# Role of Honey in Advanced Wound Care

**DOI:** 10.3390/molecules26164784

**Published:** 2021-08-07

**Authors:** Hana Scepankova, Patricia Combarros-Fuertes, José María Fresno, María Eugenia Tornadijo, Miguel Sousa Dias, Carlos A. Pinto, Jorge A. Saraiva, Letícia M. Estevinho

**Affiliations:** 1 LAQV-REQUIMTE, Department of Chemistry, University of Aveiro, 3810-193 Aveiro, Portugal; hanka.scepankova@gmail.com (H.S.); carlospinto@ua.pt (C.A.P.); jorgesaraiva@ua.pt (J.A.S.); 2 Department of Food Hygiene and Technology, Faculty of Veterinary Science, Campus de Vegazana, University of León, 24071 León, Spain; pcomf@unileon.es (P.C.-F.); jmfreb@unileon.es (J.M.F.); metorr@unileon.es (M.E.T.); 3 CIMO, Mountain Research Center, Polytechnic Institute of Bragança, Campus Santa Apolónia, 5301-855 Bragança, Portugal; miglsdias@gmail.com

**Keywords:** honey, wound-healing, antioxidant, antimicrobial, hydrogels, dermal repair, hydrogel

## Abstract

Honey is a natural product rich in several phenolic compounds, enzymes, and sugars with antioxidant, anticarcinogenic, anti-inflammatory, and antimicrobial potential. Indeed, the development of honey-based adhesives for wound care and other biomedical applications are topics being widely investigated over the years. Some of the advantages of the use of honey for wound-healing solutions are the acceleration of dermal repair and epithelialization, angiogenesis promotion, immune response promotion and the reduction in healing-related infections with pathogenic microorganisms. This paper reviews the main role of honey on the development of wound-healing-based applications, the main compounds responsible for the healing capacity, how the honey origin can influence the healing properties, also highlighting promising results in in vitro and in vivo trials. The challenges in the use of honey for wound healing are also covered and discussed. The delivery methodology (direct application, incorporated in fibrous membranes and hydrogels) is also presented and discussed.

## 1. Introduction

The skin is composed of three layers (epidermis, dermis, and hypodermis), and is considered the first physical barrier against external infectious agents. Wounds are defined as the disruption in the continuity of the skin, induced by mechanical, chemical, or thermal harms, and resulting in the loss of the defensive functions of this tissue [1].

**Figure 1:**
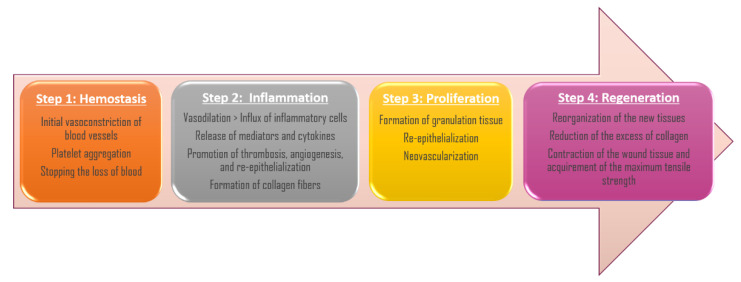
Stages of wound-healing process.

The wound-healing process has the purpose of recovering the integrity of the damaged tissue and the regeneration of the epithelium that was lost, and it is a dynamic and complex process that globally occurs in four overlapping steps: hemostasis, inflammation, tissue proliferation, and regeneration [1,2] (Figure 1).

The cascade of initial vasoconstriction of blood vessels and platelet aggregation play a key role in stopping the loss of blood. The initial vasoconstriction is followed by a vasodilation, which allows an influx of a variety of inflammatory cells which release several types of mediators and cytokines to promote thrombosis, angiogenesis, and re-epithelialization. In addition, the fibroblasts release extracellular components which initiate the formation of collagen fibers that will serve as scaffolding [1,3]. During the inflammatory phase the hemostasis, chemotaxis, and the increased vascular permeability limit further damage, close the wound, remove cellular debris and bacteria, and promote cellular migration [3]. Afterwards, the proliferative phase implicates the formation of granulation tissue, re-epithelialization, and neovascularization, a process that can last from several weeks until some months, or even more time in the case of the existence of some co-morbidities or particular patient situations [4]. In the end, during the maturation period, the new tissues are remodeled, the excess of collagen is reduced, and the wound contracts and reaches the maximum tensile strength [1].

Acute wounds derived from unexpected accidents or surgical injury commonly heal within a predictable period depending on the size, depth, and extent of damage. Nevertheless, deficiencies in the wound-healing process cause more than 38 million patients with chronic wounds worldwide, which reaches epidemic proportions and causes a large economic weight on healthcare systems [5].

Prolonged chronicity of wounds is normally related to a bacterial injured-tissue colonization, which can progress into a bacterial resistance to topical and systemic antimicrobial agents, or into biofilm development, which complicates, in both cases, their treatment [6]. In the end, this type of wound can cause sepsis and inflammation in organs and lead to increased morbidity and mortality.

The clinical considerations in wound-healing management include preventing and controlling the infection and/or contamination, maintaining the adequate moisture environment, treating edema, and preventing further injury. Conventional chronic wound care involves debridement to remove non-viable tissue and bacterial biofilms, followed by wound dressing. The common wound dressings consist of a standard cotton bandage or highly absorbent dressings, such as collagen and alginate, or hydrocolloids; however, this procedure of wound care is often ineffective. Due to this need for new, efficient, and improved therapies, there has been a revived interest in alternative treatment approaches, such as honey. The management of wound healing has become the primary field of therapeutic application of this natural product [7,8].

Honey has been used for wound healing since ancient times, mainly due to its antimicrobial activity. In addition to the broad spectrum of antibacterial activity against common wound-infecting microorganisms, honey has been demonstrated to be effective against antibiotic-resistant bacteria and was able to restore the efficacy of some antibiotics against bacteria with previously acquired resistance [8,9,10]. Furthermore, due to its several antimicrobial components and its different antibacterial action mechanisms, the development of bacterial resistance to honey is unlikely [8,11,12]. Moreover, the wound-healing ability of honey is also related to its anti-inflammatory and antioxidant activity, as well as its capacity to promote re-epithelialization and angiogenesis and stimulate skin and immune cells [13,14]. All these mechanisms act together favoring the regeneration process of the damaged tissue.

Several case studies and randomized controlled trials provide considerable evidence of the effectiveness of honey in healing different types of wounds, such as amputation wounds, burns, skin grafting sites, skin lesions, or skin ulcers including leg, varicose, malignant, diabetic, and sickle cell ulcers [15,16,17,18].

The resolution time of wounds using honey or honey-derivates varies from some days to several months depending on the type, the depth, the anatomical location, and the chronicity of the wound [17,19]. Due to its osmotic effect, honey creates a liquid layer between the dressing and the wound bed. This liquid layer is an advantage in the removal and change of wound-dressings by reducing or eliminating the pain of this process and avoiding damage of the newly grown tissue, reducing the healing time [20].

The use of honey obtained a remarkable improvement of recalcitrant wounds, and has demonstrated even more efficacy than conventional treatments using commercial wound dressings or antibiotics (systemic and topical) [19,21,22]. Honey rapidly replaces sloughs with granulation tissue and promotes a quick epithelialization and absorption of edema from around the ulcer margins, significantly reducing the healing time [19,23]. In addition, honey-based products showed excellent cytocompatibility with tissue cell cultures when compared with conventional treatments, such as silver dressings [24].

Moreover, some studies demonstrated that the combination of honey with other compounds or in combination with conventional treatment can be beneficial for diabetic foot ulcer healing, reducing the wound-resolution time, the cost of the hospital stay, and the rate of amputation when compared to other conventional treatment [17].

However, the use of honey by itself might present some limitations which are being overcome with the development of different honey formulations and honey wound dressings.

This review aims to highlight the mechanism of honey’s action in wound healing and gather the literature available regarding the use of honey and modern engineering templates for promoting modern solutions for wound and skin healing and regeneration.

## 2. The Mechanisms of Honey in Wound and Burn Healing

**Figure 2:**
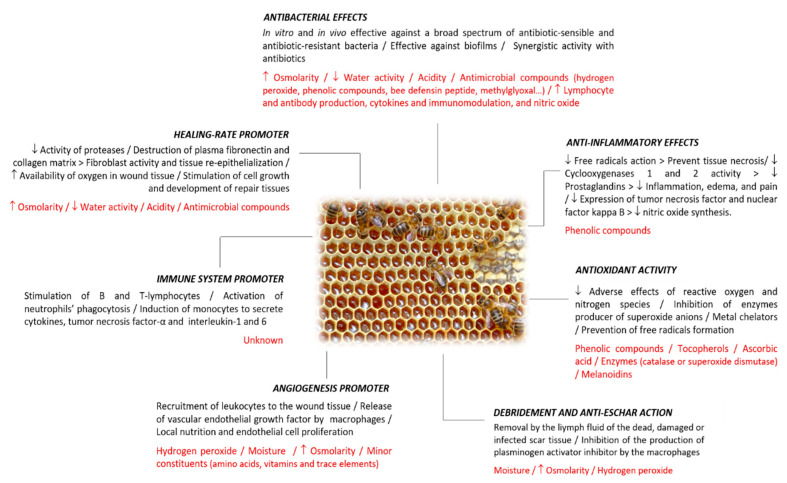
Wound healing mechanisms described for honey, different effects related to each mechanism, and the compound(s) or honey characteristics associated with the mechanism.

Honey is a natural and greatly complex substance with hundreds of compounds in its composition [8]. Honey bioactivity, and in consequence, its wound-healing potential, will be influenced by its composition, which depends mainly on the floral source and other factors, such as seasonal, environmental, as well as processing, manipulation, packaging, and storage conditions [25,26]. In addition to some inherent characteristics, such as the acidity and the osmotic pressure, the healing properties of honey in diverse types of wounds and burns have also been attributed to other components which act through different mechanisms that work together to restore the structural integrity of the damaged tissues [6,18,27] (Figure 2).

### 2.1. Antibacterial Effects

Honey has been traditionally used in the prevention and treatment of wound infections [28,29]. However, with the arrival of antibiotics, the use of honey gradually decreased. Nowadays, microbial drug-resistance has become an increasingly common concern, and honey has regained the scientific interest as an antibacterial agent [8,12,13]. Intrinsic characteristics of honey, such as high osmolarity, low water activity, and acidity, as well as some compounds, such as hydrogen peroxide, phenolic compounds, methylglyoxal, or bee defensin-1 peptide, directly affect the bacterial growth and survival [30,31,32]. In addition, honey shows an indirect antimicrobial action which involves the promotion of lymphocyte and antibody production, cytokines and immunomodulation, and nitric oxide (NO) [6,33,34,35].

Non-healing wounds, as well as burns, present an elevated risk of infection, which might increase morbidity and mortality derived from sepsis and inflammation in organs [13]. In addition, drug-resistant infections and wounds with biofilms are particularly difficult to treat, since bacteria do not respond to the therapy or are protected by a self-produced matrix of polysaccharide material [36].

Several studies have demonstrated, in vitro and in vivo, the efficacy of different varieties of honey against a broad spectrum of bacteria, including those that commonly caused wound and burn infections, such as *Staphylococcus aureus*, *Pseudomonas aeruginosa*, *Escherichia coli*, *Acinetobacter baumannii*, or *Staphylococcus epidermidis* [6,18,37,38,39,40]. In addition, honey has also been demonstrated to be effective against antibiotic-resistant bacteria [19,41,42,43], as well as against biofilms by preventing the formation and the development of the biofilm [31,44,45,46,47], by reducing the metabolic activity of already formed biofilms [44,48], or by altering the gene expression of different genes related to the formation and the development of biofilms [48,49], and is related to the bacterial quorum sensing [49,50].

Moreover, some studies demonstrated that manuka honey acts synergistically with several antibiotics, reducing the doses required to inhibit bacterial growth or reverting the antibiotic resistance previously acquired [9,10,51,52]. These results suggest a potential application of a combined therapy of honey and antibiotics.

### 2.2. Anti-Inflammatory Effects

Inflammation is the response of a living tissue to a local injury and plays a fundamental role as a defense and protection mechanism to avoid infections and to repair the affected tissue. The inflammatory phase is a necessary part of wound healing; however, when this response is not adequate, an overproduction of inflammatory mediators by immune cells, which do not respond to initial triggers, might be produced, becoming a problem for wound resolution [53]. The anti-inflammatory activity of honey is a consequence of different mechanisms.

During the inflammatory phase, the affected tissues release a high concentration of free radicals. The antioxidant compounds in honey act synergistically and can reduce the damage caused by these radicals, and therefore prevent tissue necrosis [14].

In addition, in vitro and in vivo studies have demonstrated that honey reduces the activity of cyclooxygenases 1 and 2 (COX1 and COX2) that intervene in the synthesis of prostaglandins [54,55]. Prostaglandins participate in the inflammatory response by producing vasodilation, increasing the permeability of blood vessels and allowing the passage of leukocytes, acting as an antiplatelet agent, and stimulating the nerve endings of pain. The reduction in prostaglandin concentration in plasma may induce a diminution of inflammation, edema, and pain [56].

Moreover, honey can inhibit the expression of tumor necrosis factor (TNF-α) and reduce the concentration of pro-inflammatory cytokines through the attenuation of nuclear factor kappa B (NF-κB) [54]. Furthermore, NF-κB is involved in the activation of the inducible NO synthase enzyme (iNO). During inflammation, iNO is induced by cytokines, TNF-α, interleukins, and bacterial endotoxins, producing NO.

NO is a free radical that acts as a mediator in acute and chronic inflammation and favors the healing process of tissues. However, an excess of NO or an overproduction at the wrong time can be detrimental and contribute to the development of pathologies related to inflammation [56].

Another advantage of the anti-inflammatory action of honey is the decrease in edema, thus reducing the pressure on the microvasculature of wound tissue that allows the availability of oxygen and nutrients required for growth of tissue and wound repair [20]. This effect also allows the control of the wounds’ exudate with an appropriate moisture balance, which is still a constant challenge in the healing processes [39].

The anti-inflammatory activity of honey has been mainly attributed to phenolic compounds [53,57]. However, until now, no correlation was found between the level of anti-inflammatory activity in different honey samples and the phenolic compound content [58], which might be due to the distinct types of interactions that can occur among these compounds and other compounds present in honey.

### 2.3. Antioxidant Activity

The antioxidant activity of honey is due to a wide variety of compounds, such as flavonoids, phenolic acids, tocopherols, ascorbic acid, and enzymes including catalase or superoxide dismutase [14,59,60]. In addition, melanoidins, products of the Maillard reaction, were described as the main components responsible for the radical-scavenging capacity of honey [61,62]. These substances reduce the adverse effects of reactive oxygen species (ROS) and reactive nitrogen species (RNS), inhibit the enzymes responsible for producing superoxide anions, act as metal chelators, and interfere in the chain reactions of free radicals and can play a preventative role in the process of their formation [63]. Through these antioxidant mechanisms, honey contributes to wound and burn healing by interfering with abnormal inflammatory response [6].

ROS act as messengers to give feedback amplification of the inflammatory response [20] and mediate TNF-α induced cytotoxicity [64]. Moreover, in chronic wounds, neutrophils and macrophages liberate high levels of ROS against invading bacteria [64]. The extended exposure to ROS causes cell damage of the tissue and might delay wound healing. In addition, the ROS formed in the inflammatory phase of wound healing stimulate the activity of the fibroblasts which produce the collagen fibers of scar tissue. If the inflammatory phase prolongates, it could induce hyper-granulation and fibrosis, so honey minimizes or prevents hypertrophic scarring [14,64]. In addition, flavonoids protect tissue against RNS, such as NO and peroxy-nitrite [65].

### 2.4. Debridement and Anti-Eschar Action

Wound debridement is essential in producing the functional process of tissue reparation. The conventional procedure is the surgical remotion of dead tissue, which is painful, may cause infections, and produces toxins that can destroy the surrounding tissues [6,14,27].

The moist environment produced by honey facilitates the wounds´ autolytic debridement process. The high osmotic pressure pulls out lymphatic fluid from the deeper zones, which automatically remove dead, damaged, or infected scar tissue [27,39]. In addition, lymph is a rich source of proteases that activated by the hydrogen peroxide produced when honey is diluted and assists in the debriding activity [6].

Additionally, honey inhibits the production of plasminogen activator inhibitor (PAI) by the macrophages derived from its anti-inflammatory activity [39]. PAI blocks the transformation of plasminogen, the enzymically inactive precursor of plasmin, into active plasmin. Plasmin is an enzyme that specifically digests fibrin attached to the wound surface, but does not digest the collagen matrix, which is necessary for tissue reparation, thus preventing eschar formation [20,66]. Inflammation increases the generation of PAI, so the mechanism through which honey decreases the production of PAI is probably related to its anti-inflammatory activity [20].

### 2.5. Angiogenesis Promoter

Angiogenesis occurs in the proliferative phase of wound healing. The development of new blood vessels from pre-existing ones supplies the required oxygen in the wound, which is an important stage in the healing process. This dynamic process is strongly regulated by signals from serum and the surrounding extracellular matrix environment [67]. Stimulation of angiogenesis by honey was demonstrated in an in vitro study with analogues of angiogenesis and an endothelial proliferation assay [68], and more recently, in another in vivo-model study [69].

Hydrogen peroxide (generated from glucose by the action of the enzyme glucose oxidase present in raw honey) induces the recruitment of leukocytes to wounds through a concentration gradient mechanism. Due to an oxidant induct, macrophages release vascular endothelial growth factor (VEGF), which stimulates angiogenesis [67]. In addition, the high concentration of sugars present in honey, as well as other minor constituents, such as amino acids, vitamins and trace elements, provide, in a moist environment, a local cellular energy source, which may improve local nutrition and endothelial cell proliferation [70,71,72].

On the contrary, another study has demonstrated the anti-angiogenic activity of honey is mediated by the modulation of prostaglandin E_2_ and VEGF production [73]. This disparity among studies might be explained by the honey concentration tested, since the highest pro-angiogenic effect was found in a low concentration of honey, whereas higher concentrations demonstrated anti-angiogenic activity [68].

### 2.6. Immune System Promoter

Some studies have also demonstrated the activity of honey in stimulating some immune system mediators. Honey can stimulate B- lymphocytes and T-lymphocytes and activate neutrophil phagocytosis in cell culture [20,74]. In addition, honey induces monocytes (MM6 cells) to secrete cytokines, tumor necrosis factor-α (TNF-α), interleukin-1 (IL-1), and interleukin-6 (IL-6), which activate the immune response to infection [33,34,35].

Moreover, honey stimulates antibody production during primary and secondary immune responses against thymus-dependent and thymus-independent antigens [75] and increases humoral immunity by the intrinsic NO, which activates specific signal transduction pathways in monocytes in a concentration-dependent manner [76,77].

### 2.7. Healing-Rate Promoter

In addition to all the honey effects previously described, honey acts in the regeneration of the new formed tissue, an essential step in the wound-healing process [78].

The acidification of the wound environment favors the action of macrophages, limits bacterial growth, and neutralizes the ammonia produced by bacterial metabolisms that could damage tissues [14]. However, the acidic pH of honey also limits the activity of proteases; these enzymes might inactivate the tissue growth factors and destroy the plasma fibronectin and the collagen matrix, which are necessary for fibroblast activity and tissue re-epithelialization [14,42]. In addition, the diminution of pH in the wound bed makes more oxygen available from hemoglobin in the blood [42].

Furthermore, all the nutritious components present in honey (sugars, amino acids, vitamins, and other trace elements) stimulate cell growth and the development of repair tissues [70,71,72,79,80]. In addition, re-epithelialization would also be promoted by the increment of TNF-α and interleukin-1β (IL-1β) levels. A concentration of 1% of honey has been found to stimulate the release of the cytokine TNF-α, IL-1β and IL-6 from monocytes [81], which induce the keratinocyte migration and proliferation, which are the major cellular components that are involved in the intricate mechanisms of initiation, maintenance, and completion of wound healing, and may induce collagen synthesis by fibroblasts [14,68,82].

## 3. Safety of Honey Used for Topical Treatment

The extensive scientific evidence proves that honey may offer distinct advantages over the chemotherapeutic substances currently used in the wound- and burn- healing processes. However, this natural product shows a series of limitations, and is not completely free from adverse effects.

The composition of honey is rather variable, depending primarily on the botanical origin, and secondarily on other factors such as geographical origin, or harvesting, processing, and storage conditions [38,39]. This variability determines its bioactive properties, and consequently influences the therapeutic efficacy of the wound treatment [39]. In addition, the absence of standardization and the incomplete knowledge of the active components, and the mechanisms through which they interact and act in wound healing, are the major limitations for the application of honey in medicine. For this reason, is essential to select the more appropriate varieties of honey, and it is recommended to carry out a previous screening [38].

In addition, other considerations must be considered before honey application in wounds. The low pH, derived from the presence of organic acids in honey, may contribute to a stinging or burning sensation when it is applied to a damaged tissue [83]. Besides this unpleasant sensation, it is necessary to consider that, although minor, there is a risk of wound infection, mainly related to the presence of clostridial spores which have occasionally been found in honey [39,84]. This risk can be reduced by using gamma-irradiation, which inactivates the spores without modifying the original biological activity [85,86]. Nevertheless, no cases of wound infection due to clostridial spores related to the use of non-irradiated honey on wounds have been reported to date.

Furthermore, the honey used for medical purposes must be free of any chemical contamination, such as pesticides, herbicides, or heavy metals. In this sense, to guarantee the maximum purity, honey should be collected in areas that meet the requirements for organic production, as well as following rigorous quality, processing and storage standards [8,19]. In addition, is necessary to consider that some varieties of honey might present toxic active compounds originating from the nectar of species such as rhododendron, oleanders, mountain and sheep laurels, or azaleas. However, these effects have been described by honey ingestion [87,88].

## 4. Biomedical Application of Honey in Advanced Wound Care

### 4.1. Medical Grade Honey and Honey Ointments for Topical Application

The safety threats previously described in wound treatment with honey are overcome by medical-grade honey approved for wound care [18,39]. Indeed, the medical-grade honeys are sterilized by gamma irradiation with the aim to kill Clostridium spores, produced under rigorous standards of hygiene, without pollutants or contaminating pesticides in its composition, and standardized under different defined criteria [8]. They have potent in vitro bactericidal activity against antibiotic-resistant bacteria and are approved for application in wound management. Having reproducible antibacterial activity, these honeys are produced under controlled conditions in greenhouses (i.e., Revamil source honey); medical grade Leptospermum-derived Manuka honeys are analyzed individually by each batch to assess the Unique Manuka Factor (UMF) that gives a number based on its bactericidal activity [89]. Unlike other varieties, the antibacterial activity of Manuka honey is based on its non-peroxide activity related to compounds which are mainly present in this variety, such as methylglyoxal, leptosperin, or methyl syringate [38].

Medical-grade honey can be directly applied to the wound bed and then covered with conventional dressing. However, in high-exuding wounds, honey can become less viscous and diluted. The liquid state of honey might complicate its application and its permanence on wound and burn environments, and in the treatment of excessively exudative lesions, honey might be diluted to concentrations that present minimal or no effects in short periods of time [7,39]. Nevertheless, according to [6], even when honey is heavily diluted by wound exudate, it will still have potent enough antibacterial activity to inhibit the growth of bacteria (the MIC values were found to be below 11%).

In clinical practice today, several honey-based wound-care commercial preparations in the form of gels, ointment, and dressings, are approved by the US Food and Drug Administration (FDA) and registered as medical devices [90]. Most of them are formulated with medical-grade Manuka honey, since it is one of the most studied varieties of honey in the world, and it was the first honey type to obtain the status of medical-grade honey. However, there are also alternatives that use other types of honey, such as buckwheat, multifloral, and Revamil source honey, among others [8]. For instance, a honey-based gel formula can be prepared with 100 % medical-grade honey without the addition of other ingredient (Table 1) or can be mixed with other agents(s) such as natural emollients (e.g., lanolin, polyethylene glycol, glycerine, myristil myristrate) or different plant waxes and gelling agents [90].

The FDA-approved honey-based devices are indicated in the treatment of different types of wounds, such as low and moderate-to-heavy exuding wounds, diabetic foot ulcers, leg ulcers, pressure ulcers, burns, traumatic wounds, surgical wounds, chronic wounds, or colonized acute wounds, among other indications [18]. Despite the availability of these products, their use in medical practice is still limited, probably due to the misconception that there is no evidence to support the use of honey with therapeutic purposes, as well as the scarce promotion and diffusion of honey products for wound care [20].

Nevertheless, effectiveness of the Medihoney^TM^ Antibacterial Wound Gel has been evaluated in eight post-coronary artery bypass graft (CABG) patients. The gel was selected as the active primary product of choice for all the graft wounds (seven of the eight patients had wound infections). The honey-based gel was applied directly onto the wound bed and covered with an adhesive-bordered non-adherent gauze dressing. The wounds all reduced in size and there was a significant reduction in pain, odor, and exudate. Moreover, the wound gel had reduced the bioburden of the wounds enabling them to progress to healing. Finally, the use of medical honey became a regular dressing choice within the authors’ cardiothoracic unit [91]. Moreover, the combined treatment with honey-based gels (L-Mesitran Soft) and ointment (L-Mesitran Ointment) has been successfully applied for treatment of infected ulcers in diabetics patients [19]. Indeed, the use of ointments help to entrap water, keep the skin moist, and provide an emollient protective film, which are all crucial elements for wound healing [19,90], while the layer of gel fights infections and optimizes wound healing [19,90].

The medical-grade honey, as well as the ointments and gels, is applied to the wound bed and requires a secondary conventional dressing (e.g., cotton wool bandage) to contain the honey in the wound bed environment, which on removal cause pain [92].

Therefore, recent research focuses on the development of different materials or matrices to convey honey, control its delivery, and act as absorbent secondary dressings [7,39,93,94,95].

### 4.2. Honey-Based Advanced Wound Care Products

Many years ago, wound management was based on covering the wound using conventional dressings (i.e., gauzes, absorbent cotton, bandages). However, they are limited in terms of influencing/accelerating wound healing and preventing/treating infections. Currently, wound management has been updated due to a greater understanding of the molecular and cellular processes involved in wound healing. Additionally, with advancements in technology, the design and functionality of wound dressings has advanced in the direction of multi-functionality [96]. The modern dressings are designed to maintain the moist wound environment and promote healing [97]. Moreover, the critical necessities of modern wound dressings include biocompatibility, no cytotoxic effects, a rate of biodegradability directly proportional with the rate of formation of new tissue, a release of incorporated bioactive compounds (drugs), and the control of possible infections [96].

Tissue engineering has recently introduced wound dressings/scaffolds as an alternative treatment of wounds with advanced properties, suitable for keeping a moist environment while absorbing exudates, creating a barrier against pathogens, and facilitating drug delivery systems [96].

The recent in vitro and in vivo research demonstrated that honey is a valuable addition to many tissue-engineering templates in eliminating bacterial infection, aiding in inflammation resolution and improving tissue integration with the template (Table 2 and Table 3) [28]. Currently, hydrogels and electrospun nanofibers are the most researched types of honey-incorporated scaffolds [98].

#### 4.2.1. Honey-Based Hydrogels

Hydrogels are high-water-content materials prepared from cross-linked polymers, such as chitosan, and can provide sustained, local delivery of a variety of therapeutic agents [106]. Incorporation of honey into the hydrogel system can beneficially affect the water absorption capacity of the polymer (of the hydrogel) and increase the antibacterial activity of the scaffold [99].

Noori, Kokabi, and Hassan (2018) investigated a honey-loaded PVA/chitosan/montmorillonite nanocomposite (PCMH) hydrogel dressing as a drug model for wound healing. Results demonstrated the ability of the PCMH nanocomposite hydrogel to smart release honey against pH and temperature changes. The maximum release of honey from the hydrogel occurred at pH 7, while the minimum was at pH 2. Independently of the pH, increasing temperature caused higher honey release from the hydrogel matrix. However, the addition of the nanoclay (montmorillonite) to the hydrogel decreases the hydrogel swelling and delays the honey release. Nevertheless, the authors suggested that the honey-loaded nanocomposite hydrogel could be used in low exudate wounds to supply optimized humidity in the wound bed [102].

Different honey concentrations have different efficacies in the scavenging of free radicals and promoting epithelial cell proliferation [119]. It has been shown that the release of honey increases when increasing its concentration in hydrogel, regardless of the polymeric hydrogel used in the formulation. For instance, the honey 75%-chitosan formula showed the best healing properties (regeneration of the epidermis tissue and the formation of new blood capillaries) compared to honey hydrogel formulae with a lower concentration of honey (up to 50%, *w*/*w*). Similarly, an 80%-Manuka/PVA wound dressing hybrid hydrogel showed sustained release of honey over 24 h with progressively low adhesion to the wound bed that protects new epithelialization and promotes cell proliferation. Both dressings demonstrate the high value of cell viability and proliferation and promoted antibacterial activity, being suitable for wounds with moderate to relatively high exudate [100].

Further, one of the advantages of using hydrogels instead of conventional designs is their transparency, which allows us to observe the status of the burn or wound without removal of the dressing [103]. In vivo study showed that burns treated with honey hydrogel sheets were completely healed after 12 days with intact epidermis and topical proliferation of hair follicles. In contrast, burns treated with commercial ointment (MEBO-treated burns) and non-treated burns presented 15% and 63% unclosed wound areas, respectively [104].

In addition, hydrogel wound scaffolds containing honey do not function merely as coverage to provide a clean, moist environment for healing, but also directly contribute to enhanced tissue regeneration and recovery [104].

#### 4.2.2. Honey-Based Electrospun Nanofiber Scaffolds

In biomedical applications, the nanofiber membranes prepared by electro-spinning are used in wound dressings, biosensing, tissue engineering scaffolds, artificial organs, and drug delivery [114]. The formulation of the electrospun nanofiber scaffolds contain protein-based polymers, such as collagen, gelatin, and silk, or/and polysaccharide-based polymers such as chitosan, hyaluronic acid, and alginate. Prepared stirred polymeric solution is loaded into a syringe that is attached to a needle of which the tip exhibits voltage [120].

The advantageous properties of nanofibers are a large surface area to volume ratio, high porosity, and a very small pore size, which lead to high exudate absorption, better wound permeation, and prevention of further infection [112].

The fabrication of honey-based electrospun nanofibers increases interest due to the enhanced activity realized upon combining the advantages of the nanofibrous structure, primarily the increased surface-to-volume ratio with the advantageous properties of honey (Table 3) [112].

Wound healing scaffolds are expected to absorb body fluids and maintain hydration, but without increasing infection of the biofilm. Indeed, wounds with biofilm fail to re-epithelialize, show vascular granulation tissue, and consist of recalcitrant microbes. Honey/PVA nanofiber membranes were found to effectively decrease the biofilm formation [113]. Manuka honey is effective against both Gram-positive and Gram-negative bacteria; however, results of honey scaffolds containing 1–20% Manuka/PCL nanofibers indicated that the controlled release of smaller amounts of honey by the scaffold is more effective against Gram-negative-bacteria-infected wounds. Interestingly, opposite results have been found for high-concentration honey chitosan electrospun nanofibers. The honey/PVA/chitosan membrane (30%:7%:3.5%) enhanced antibacterial activity against *S. aureus* (complete inhibition after 48 h with 30%: 7%:5.5 %) and showed poor antibacterial activity against the Gram-negative *E. coli*. Moreover, the scaffold showed high biocompatibility and low cytotoxicity effects [108].

## 5. Conclusions

The use of honey for biomedical applications has gained special focus over the years, with the development of novel applications for this natural product, taking advantage of its unique chemical characteristics. Due to its characteristics, namely, low pH and water activity, it presents a good microbiological, enzymatic and (bio)chemical stability, which can be lost if not properly processed for safe use by means of keeping both functionality and microbiological safety. Thus, the design of proper honey processing methodologies is of utmost importance for its use. As reviewed, honey presents a very promising potential to be used in wound-healing processes, either by direct application, incorporated in fibrous membranes, or in hydrogel, with very promising results in either in vitro and in vivo trials. Nonetheless, further research is needed to overcome the main challenges on the use of honey for biomedical applications.

## Figures and Tables

**Table 1:** Commercially available medical-grade honey and honey-based gels and ointments for wound healing.

Product Name	Product Type	Composition
Manuka Guard^®^ Medical Grade Manuka Honey	Honey	100% Manuka honey
Manuka Fill^®^	Honey	100% Manuka honey
Ectocare^®^ Manuka Fill^TM^	Honey	100% Manuka honey
ManukaDress–T	Honey	100% Manuka honey
Activon^®^ Tube	Paste formula	100% Manuka honey
Manuka Health^®^ Wound Gel	Gel formula	94% Manuka honey with natural gelling agents
Medihoney^®^ Barrier Cream	Cream formula	30% Manuka honey, other non-described components
Medihoney^®^ Gel Wound & Burn Dressing	Gel formula	100% Manuka honey in a hydrocolloidal suspension
Melladerm^®^ Plus	Gel formula	45% medical-grade multi-flower honey, other non-described components
Melloxy^®^	Gel formula	40% medical-grade multi-flower honey, 11% ozonated vegetable olive oil, other non-described components
MANUKApli^®^	Gel formula	100% Manuka honey
L-Mesitran^®^ Soft	Gel formula	40% medical-grade honey (not Manuka) with lanolin, polyethylene glycol, and vitamins C and E
L-Mesitran^®^ Ointment	Gel formula	48% medical-grade honey (not Manuka), lanolin, cod liver oil, sunflower oil, calendula, aloe vera, zinc oxide, and vitamins C and E
Revamil Gel^®^	Gel formula	100% medical-grade honey (not Manuka)
Revamil Balm^®^	Balm formula	25% medical-grade honey (not Manuka), arachis oleum, cera alba, glyceryl oleate, aqua.
Surgihoney™RO^®^	Gel formula	Mixture of medical-grade honey from various sites/floral sources engineered to produce hydrogen peroxide and reactive oxygen species when diluted in water
Therahoney^®^ Gel	Gel formula	100% Manuka honey

**Table 2:** Examples of honey hydrogel-based wound dressings, methods of formation, evaluated models and main findings.

Honey Hydrogel Formulation			In Vivo Wound and Burn Model			Findings	References
Incorporated Honey	Hydrogel Matrix	Method of Hydrogel Formation	Type of Mice	Location of Lesson	Type of Wound/Burn		
6% (*v*/*v*) Gelam *(Melaleuca Apis melífera)* honey (Malaysia)	PVP 15% (*w*/*v*), PEG 1% (*v*/*v*), protein-free agar solution 1% (*w*/*v*).	Electron beam irradiation (25 kGy)	96 male Sprague Dawley rats	Dorsum of rat	Deep partial thickness burn wounds	Good transparency; slightly acidic (pH 4.3); high capabilities in absorbing fluid. Significant acceleration of dermal repair and advanced re-epithelialization. Modulation of proinflammatory cytokines in wound healing. Synergistic effect of hydrogel matrix and incorporated honey.	[99]
Up to 3.5 % (*w*/*w*) Iran honey	PVA 10 % (*w*/*w*), CM-chitosan up to 3.5 % (*w*/*w*), water 85 % (*w*/*w*).	^60^Co Gamma-ray (radiation method) up to 40 kGy; ^60^Co Gamma-ray followed by 3 cycles of freeze–thawing (combinational method).	male NMRI mice	Dorsum of mice	0.7 cm × 0.7 cm wound	Acceptable swelling degree, transparency and inhibition of the growth of *E. coli* bacteria. The hydrogel containing more honey in its formulation has a more effective action in the wound healing process of the mouse. The mechanical strength of the hydrogels prepared by the combinational method was higher than by radiation method.	[100]
70% honey-based alginate hydrogel	Alginate hydrogel	-	20 male Wistar rats	Side of vertebral column between the ears	Full-thickness wound (1 cm × 1 cm)	Epiderm growth (after 21 days) and collagen synthesis (after 14 days). Wound-healing influences were attributed to the synergistic effect of the alginate hydrogel and the incorporated honey.	[101]
15 wt% PVA/chitosan nanoclay hydrogel	PVA 10% (*w*/*w*), Chitosan 2% (*w*/*w*), TPP (chitosan crosslinking agent), Montmorillonite up to 3 wt%, Acetic acid solution 2% (*v*/*v*)	Freeze–thawing method (freezing at −15 °C/24 h and subsequently thawing at room temperature for 24 h).	15 female mice	Dorsum	Full-thickness wound (1 cm × 1 cm)	Honey-loaded hydrogel nanocomposite wound dressings (PCMH) had better wound-healing ability than nanoclay hydrogel without honey (PCM) and hydrogel without nanoclay and honey (control group). The wound size reduction at the third post-operation day was: 39.62% (control), 39.62% (PCM), and 39.62% (PCMH); at the sixth day: 55.23% (control), 58.38% (PCM), and 72.60% (PCMH).	[102]
10% and 20% (*v*/*v*) Chicory honey (Iran)	Chitosan, gelatin 5% (*w*/*v*), PVA 10% (*w*/*v*), acetic acid 3% (*w*/*v*); Ratio of 2:1:1 (*v*/*v*/*v*) chitosan, PVA, gelatin solution	3 cycles of freeze–thawing (freezing at −20 °C for 20 h, and subsequently thawing at 25 °C for 4 h)	18 male Wistar rats	Back of rats	Full thickness excisional wounds (2 cm × 2 cm)	The higher concentration of honey in the hydrogel facilitated the wound-healing process from inflammation to proliferation, and finally, to the maturation phase. Almost 50% wound closure was observed after 4 days (20% *v*/*v* honey); and 95% after 12 days (10 and 20% *v*/*v* honey).	[103]
20 g of diluted (50:50 *w*/*w*) raw sunflower honey (China)	Chitosan, gelatin, honey (ratio of 0.5:20:20, *w*/*w*/*w*). Distilled water up to 100 g (final volume).	Standing and cooling to room temperature. Sterilization of hydrogel sheets with UV rays for 45 min.	4 male rabbits	Dorsum	Second degree burn wound (3 cm × 3 cm)	On day 12, the burns treated with honey hydrogel sheets (HS) were completely healed with intact epidermis and topical proliferation of hair follicles. In contrast, MEBO-treated burns (commercial ointment) and non-treated burns presented 15% and63% unclosed wound area, respectively.	[104]
Solution (1:1, *v*/*v*) of liquid Manuka honey (New Zealand)	pectin	Hot-air-dried at 40 °C and conditioned in air drier at 25 °C for 5 days. Sterilization by gamma-irradiation at 25 kGray	36 male Sprague Dawley rats	Dorsum	Full thickness excisional wounds (2 cm × 2 cm)	Topical administration of pectin and pectin-honey hydrogels accelerate wound healing in rats. On the 23rd day, the entire surface of the lesion treated with the dressing was covered with hair follicles and matured fibrous tissue.	[105]
Honey (Egypt) Up to 75% (*w*/*w*)	Carbopol 934, Chitosan, Methyl paraben, TEA, GAA, purified water.	Cold mechanical method (placed in refrigerator)	10 albino mice	Dorsum	Third-degree burn type with a focal wide area of necrosis in the epidermis	Honey 75%-chitosan formula showed the best healing properties (regeneration of the epidermis tissue and the formation of new blood capillaries) compared to the pure honey and the commercial product tested (silver sulphadiazine).	[106]
Manuka honey 80% *w*/*w*)	PVA, borax (crosslinking agent)	Solution was molded in Petri dishes and kept at 50 °C overnight.	-	-	-	A wound dressing hybrid hydrogel with sustained release of honey over 24 h and with progressively low adhesion to the wound bed that protects new epithelialization and promotes cell proliferation. Antibacterial activity observed against the tested S. aureus.	[107]

**Table 3:** Examples of honey electrospun nanofibrous scaffolds, obtention methods and main findings.

	Composition		Crosslinking of Fibres			Findings		
Nanofibrous Honey Scaffold	Honey	Material	Method	Conditions		Wound Healing Properties	Characteristics	References
**Honey/PVA/** **Chitosan**								
High concentration honey chitosan electrospun nanofibers (HP-chitosan)	Honey (20–40%)	chitosan (1.5% to 5.5%), PVA.	Chemical crosslink Physical crosslink-heating and freeze/thawing	GA vapors heating (under vacuum in an oven, up to 110°/up to 24 h) and freezing (in liquid nitrogen)/thawing (at room temperature.	In vitro	HP–chitosan; (30%:7%:3.5%) enhanced antibacterial activity against *S. aureus* (complete inhibition after 48 h with 30%:7%:5.5 %), poor antibacterial activity against *E. coli.*	HP–chitosan (30%:7%:3.5%) upon aging for more than 2 days acquired the optimum viscosity required for easy spinning and formation of uniform nanofiber. Effective biocompatible wound dressing.	[108]
Honey /PVA/chitosan nanofibers	Honey (10–30%)	chitosan (3.5%), PVA (7%), acetic acid (1%).	Chemical crosslink Heating	GA vapors 40 °C	In vitro	Enhanced antibacterial activity against Gram-positive *S. aureus* over the Gram-negative *E.coli*	Increase in fiber diameter; Large pore diameter reaching 140 μm (10, 30% honey); Degradation decreased with crosslinking of the fibre mats.	[109]
Honey-PVA-chitosan nanofibers green wound dressing (bio-compatible apitherapeutic nanofibers international patent (2006.01)	Honey (25–50%)	PVA, chitosan (1.5–11%) bee venom, propolis, garlic (2–30%), bacteriophage			In vitro	Honey-PVA-chitosan nanofibers loaded with bee venom/bacteriophage exhibited potent antibacterial activity against Gram-positive and Gram-negative strains and achieved nearly complete killing of multidrug-resistant *Pseudomonase aeruginosa*. Enhanced wound healing and improved biocompatibility		[110,111]
**Honey/PVA**								
Honey, pomegranate peel extract and bee venomnanofibrous wound dressing	Manuka honey (MH) (10–25%), lyophilized multiflora honey powder (25 %)	PVA (up to 12%), bee venom (BV, 0.01%), methanolic pomegranate peel extract (PPP, up to 2.5%).	Chemical crosslink Heating	25% GH vapours in a vacuum oven at 40 °C/24 h	In vivo	MH/PPP/BV/PVA (25%/2.5%/0.01%/9.7%) close resemblance to normal skin at day 10;	**No cytotoxic** (100 % viability, tested on L929 fibroblast cells)	[112]
					In vitro	effective inhibition of bacterial growth for *S. aureus* and *E. coli.*		
PVA/honey nanofibers	Iran-Tabriz honey (up to 40%)	PVA, dexamethasone sodium phosphate (anti-inflammatory drug loaded up to 15%)	Only electro-spinning				Decreased diameter of electrospun fibers caused by increasing honey concentration.	[104]
PVA/honey nanofibrous scaffolds (with low honey concentration for internal tissue regeneration)	Dabur honey (India) (0.2–1% *w*/*v*)	PVA 12% (*w*/*v*)	Chemical crosslink	GA vapours (2 M) for 24 h.	In vitro	Drastically reduced biofilm Nanofiber membranes with 0.5% honey loading can be suggested as optimum concentration	Minimal weight loss of fibers for 10 days.	[113]
PVA–DES–honey nanofibers	Acacia honey (China) (5%, *w*/*v*)	PVA (8%, *w*/*v*), DES (5%, *w*/*v*)	Only electro-spinning		In vitro	Possess excellent antimicrobial activity (*E. coli, S. aureus*) total bacterial reduction of 37.0% and 37.9% against *E. coli* and *S. aureus,* respectively, after 6 hour incubation in bacterial cultures; excellent cytocompatibility, non-toxic	The nanofiber materials dissolved rapidly in artificial saliva solutions, suggesting potential use of materials for fast-dissolving drug delivery in oral cavities	[114]
					In vivo	PVA–DES–honey nanofibers accelerated the wound healing process, and improved the wound healing rate on rat skin to 85.2% after 6 days of surgery, when compared to the control PVA (68.2%) and PVA–DES (76.3%) nanofibe		
**Honey/PICT**								
PICT/honey nanofibrous	Pakistan forest honey (10–20%)	PICT					Good elastic behavior and tensile strength (PICT/honey nanofibers containing 15% honey); good releasing efficiency, complete release of honey in 15 min, the maximum release in 10 min (72 mg/L, 56% of honey).	[115]
**Honey/silk fibroin**								
Honey-silk fibroin (SF) electrospun scaffold	Medical grade Manuka honey (Melita) (5% of 5 and 20 UMF)	Lyophilized SF (5%)			In vitro	Tissue engineered scaffold could be incorporated with MH of any UMF, resulting in the same bactericidal outcome	No significant difference in porosity, bacterial clearance and adhesion, glucose release, or proliferation of cells as effected by the incorporation of 5 versus 20 UMF MH.	[116]
MH/SF composite fibrous matrices manufactured by green electrospinning	Manuka honey (UMF 5+) 0, 10%, 30%, 50%, 70%	SF 20% (*wt*/*v*) and 2% (*wt*/*v*) PEO			In vivo	The addition of MH improved the wound healing rate of the SF fibrous matrices; wound treated with MH (70%)/SF showed a similar healing effect as the AquacelAg dressing.	Excellent biocompatibility m (incorporation of MH could further improve the affinity of SF fibrous matrices for cells)	[117]
					In vitro	Increase in the bacterial inhibition efficacy with increasing the content of MH.		
**Honey/PCL**								
Manuka honey-PCL nanofiber scaffolds	1, 5, 10, and 20% *v*/*v* Manuka honey solutions	15 wt% of PCL (Polycaprolactone)				Honey scaffolds demonstrated significant clearance in only the Gram-negative *E. coli*	Lower elasticity and strength with honey incorporation, but showed no notable change in material degradation rate with the presence of honey over a 28 day PBS soak.	[118]

## Data Availability

The data presented in this study are available on request from the corresponding author.
